# Die interdisziplinäre alterstraumatologische Visite: Empfehlungen für den Klinikalltag

**DOI:** 10.1007/s00113-020-00833-3

**Published:** 2020-06-12

**Authors:** J. Heck, A. Ranker, A. Wranke, L. Kano, C. Macke, J. Rademacher, D. O. Stichtenoth, O. Krause

**Affiliations:** 1 Institut für Klinische Pharmakologie, Medizinische Hochschule Hannover, Carl-Neuberg-Straße 1, 30625 Hannover, Deutschland; 2 Klinik für Unfallchirurgie, Medizinische Hochschule Hannover, Hannover, Deutschland; 3 Klinik für Rehabilitationsmedizin, Medizinische Hochschule Hannover, Hannover, Deutschland; 4 Klinik für Gastroenterologie, Hepatologie und Endokrinologie, Medizinische Hochschule Hannover, Hannover, Deutschland; 5 Institut für Allgemeinmedizin, Medizinische Hochschule Hannover, Hannover, Deutschland; 6 Klinik für Pneumologie, Medizinische Hochschule Hannover, Hannover, Deutschland; 7 Zentrum für Medizin im Alter, DIAKOVERE Henriettenstift, Hannover, Deutschland

**Keywords:** Patientenbehandlung, Traumazentren, Pharmakotherapie, Delir, Antimicrobial Stewardship, Patient care, Trauma centers, Drug therapy, Delirium, Antimicrobial stewardship

## Abstract

**Hintergrund:**

Ein orthogeriatrisches Komanagement kann die Behandlungsqualität alterstraumatologischer Patienten verbessern.

**Fragestellung:**

Ziel dieser Arbeit ist es, Handlungsempfehlungen für den Klinikalltag zu formulieren, um die stationäre Behandlung unfallchirurgischer geriatrischer Patienten zu verbessern.

**Material und Methoden:**

Über einen Zeitraum von 7 Monaten wurden 226 Patienten an 29 definierten, wöchentlichen Tagen unter Berücksichtigung aktueller Laborergebnisse, Vitalparameter, der Medikation sowie der klinischen Einschätzung des Pflegepersonals besprochen und visitiert. Neben Ärzten verschiedener Fachrichtungen (Unfallchirurgie, Geriatrie, Klinische Pharmakologie, Mikrobiologie) nahmen Pflegekräfte und Case Manager an den Visiten teil.

**Ergebnisse:**

Pro Patientenkontakt wurden im Durchschnitt drei Handlungsempfehlungen ausgesprochen (zwei pharmakologische und eine allgemeine Empfehlung [z. B. zum Flüssigkeits- und Delirmanagement]). Pharmakologische und allgemeine Empfehlungen wurden in verschiedene Untergruppen eingeteilt. Die häufigste pharmakologische Empfehlung war, ein Medikament abzusetzen (30,4 % aller pharmakologischen Empfehlungen).

**Diskussion:**

Bei der Pharmakotherapie geriatrischer Patienten müssen Kontraindikationen, Nebenwirkungen, Doppelverordnungen, zirkadiane Aspekte und die Nierenfunktion beachtet werden. Durch regelmäßige Reevaluation medizinischer Fremdkörper kann katheterassoziierten Infektionen vorgebeugt werden. Das Erkennen und die Behandlung eines postoperativen Delirs sind essenzielle Bestandteile einer interdisziplinären alterstraumatologischen Visite. Zur Beurteilung einer antiinfektiven Therapie leistet die Expertise eines Mikrobiologen/Infektiologen einen wertvollen Beitrag.

## Abstract (en)

**Background:**

An orthogeriatric co-management can improve the quality of care for geriatric trauma patients.

**Objective:**

The aim of this study was the establishment of treatment recommendations for the clinical routine in order to improve the quality of care for geriatric trauma patients.

**Material and methods:**

Over a period of 7 months, 226 patients were discussed and visited once a week on 29 defined days, taking into account current laboratory results, vital signs, the medication as well as the clinical assessment by the nursing personnel. Besides physicians of different medical specialties (trauma surgery, geriatrics, clinical pharmacology, microbiology), members of the nursing staff and case managers took part in the ward rounds.

**Results:**

On average, three treatment recommendations were made per patient visit (two pharmacological and one non-pharmacological recommendation [e.g. concerning fluid and delirium management]). The pharmacological and non-pharmacological recommendations were divided into several subcategories. The most frequent pharmacological recommendation was the discontinuation of a drug (30.4% of all pharmacological recommendations).

**Conclusion:**

The pharmacotherapy of geriatric patients requires careful consideration of contraindications, adverse drug reactions, duplicate medications, circadian aspects, and renal function. Regular re-evaluation of medical equipment can prevent catheter-associated infections. Identification and management of postoperative delirium is an integral component of the interdisciplinary orthogeriatric ward round. Evaluation of anti-infective treatment regimens with the expertise of a microbiologist/infectiologist proved to be very beneficial.

Durch den demografischen Wandel nimmt die Zahl geriatrischer Patienten auf unfallchirurgischen Stationen stetig zu. Geriatrische Patienten stellen aufgrund von Komorbiditäten sowie der oft bestehenden Polypharmazie eine besondere Herausforderung dar. *In dieser Arbeit wird aufgezeigt, welche Probleme bei geriatrischen unfallchirurgischen Patienten auftreten, und wie diese mittels medikamentöser und nichtmedikamentöser Therapievorschläge adressiert werden können. Es sollen klinisch-praktische Empfehlungen gegeben werden, um Unfallchirurgen bei ihrer Visite am Krankenbett zu unterstützen.*

## Hintergrund

Die Alterstraumatologie beschäftigt sich mit der interdisziplinären Versorgung von Frakturen, die eine hohe Prävalenz bei älteren Menschen haben. Hierzu zählen u. a. Hüft‑, Wirbelkörper- und proximale Humerusfrakturen [2]. Vor allem Hüftfrakturen gehen bei alten Menschen mit einer erhöhten Mortalität einher [11]. Die mit einem hohen Lebensalter assoziierten Risikofaktoren Sarkopenie, *Frailty* und Schwindel erhöhen die Sturzneigung und tragen entscheidend zur hohen Prävalenz der oben genannten Frakturtypen bei [5]. In gleicher Weise steigern sie das Risiko für (post)operative Komplikationen und können dazu führen, dass die Mobilität der Patienten dauerhaft eingeschränkt bleibt, bis hin zur Pflegebedürftigkeit.

Patienten in höherem Lebensalter leiden in der Regel an diversen Vorerkrankungen, verfügen über eine umfangreiche Hausmedikation und weisen ein erhöhtes Risiko für die Entwicklung eines postoperativen Delirs auf [5]. Daher gestaltet sich die stationäre Behandlung geriatrischer Patienten in der Unfallchirurgie äußerst komplex. Es gibt umfangreiche Studiendaten, dass eine internistisch-geriatrische Mitbetreuung (sog. orthogeriatrisches Komanagement) alterstraumatologischer Patienten das Behandlungsergebnis im Vergleich zu einer rein unfallchirurgischen Behandlung im Hinblick auf die Komplikations- und Mortalitätsrate, das funktionelle Ergebnis und den Erhalt der Selbstständigkeit verbessert [14, 15]. Zentraler Bestandteil ist hierbei die Behandlung geriatrischer Patienten im multidisziplinären Team: Neben der Kooperation von Unfallchirurg und Geriater ist die Einbeziehung von Pflegekräften, Physiotherapeuten etc. entscheidend [5]. Darüber hinaus entwickeln sich zunehmend interdisziplinäre Visitenmodelle, bei denen auch Klinische Pharmakologen sowie Infektiologen/Mikrobiologen den Unfallchirurgen am Patientenbett beraten.

## Fragestellung

Ziel dieser Arbeit ist es, anhand eines repräsentativen alterstraumatologischen Patientenkollektivs einer deutschen Universitätsklinik Handlungsempfehlungen für den Klinikalltag abzuleiten, um hierdurch einen Beitrag zur Verbesserung der stationären Behandlung unfallchirurgischer Patienten in höherem Lebensalter zu leisten. Hierzu wurden die während unserer interdisziplinären alterstraumatologischen Visiten ausgesprochenen medikamentösen und nichtmedikamentösen Handlungsempfehlungen retrospektiv ausgewertet.

## Methodik

### Stichprobe

Die Untersuchungsstichprobe bestand aus insgesamt 226 Patienten, die an 29 Visitentagen im Einwochenturnus über den Zeitraum 31.05.2019–13.12.2019 untersucht wurden. Die Datenauswertung erfolgte retrospektiv in anonymisierter Form. Die Patienten wurden auf 4 Stationen der Klinik für Unfallchirurgie der Medizinischen Hochschule Hannover visitiert und mussten neben der vollstationären Behandlung und einem Alter ≥65 Jahre mindestens eines der folgenden Charakteristika aufweisen: geriatrietypische Multimorbidität, Polymedikation (Einnahme ≥5 Arzneimittel), Verdacht auf eine nosokomiale Infektion bzw. bereits bestehende antiinfektive Therapie, erhöhtes Risiko für die Entwicklung eines postoperativen Delirs. Es existierten keine spezifischen Ausschlusskriterien. Im Mittel (± Standardabweichung (SD)) wurden 7,8 ± 2,7 Patienten/Visitentag untersucht (Spannbreite: 4 bis 14 Patienten).

### Ablauf der interdisziplinären alterstraumatologischen Visite

Neben den behandelnden Unfallchirurgen nahmen an der Visite eine Pflegekraft, eine Assistenzärztin aus der Inneren Medizin, ein Facharzt für Innere Medizin und Geriatrie, eine Fachärztin des Antibiotic-Stewardship(ABS-)Teams, ein Assistenzarzt aus der Klinischen Pharmakologie sowie eine Case Managerin teil.

Die für die Visite ausgewählten Patienten wurden vorbesprochen und anschließend unter Berücksichtigung aktueller Laborergebnisse, der Vitalparameter sowie der aktuellen Medikation visitiert. Im Rahmen der klinischen Visite wurden die Patienten befragt (z. B. nach Schmerzen, Atembeschwerden etc.) sowie Orientierung und Vigilanz klinisch-orientierend überprüft. Des Weiteren wurden eine symptombezogene körperliche Untersuchung durchgeführt, die Operationswunde(n) inspiziert und medizinische Fremdkörper wie Drainagen, periphere (PVK) und zentrale Venenkatheter (ZVK) und Harnblasenkatheter auf ihre Indikation und mögliche Entzündungszeichen hin kontrolliert. Die aktuelle Medikationsliste aus der Patientenkurve (Papierformat) wurde mithilfe des elektronischen Arzneimittelinformationssystems AiD Klinik® (Dosing GmbH, Heidelberg, Deutschland) auf Doppelverordnungen, die Korrektheit der Dosierungen, mögliche pharmakokinetische und -dynamische Wechselwirkungen sowie auf für ältere Menschen potenziell inadäquate Medikamente gemäß der PRISCUS-Liste [8] analysiert. Darüber hinaus wurde die Einleitung bzw. Fortführung einer antiinfektiven Therapie unter Berücksichtigung mikrobiologischer Untersuchungsergebnisse besprochen. Sozialmedizinische Aspekte wie die Weiterbehandlung nach Abschluss des stationären Aufenthalts oder die Beantragung häuslicher Unterstützung wurden ebenfalls diskutiert.

### Statistik

Für die statistische Auswertung wurde das Programm Microsoft® Excel® 2010 (Redmond, Washington, USA) verwendet. Die Darstellung der Ergebnisse erfolgt deskriptiv mittels Mittelwert, Median, Standardabweichung und Spannbreite für numerische Parameter. Für dichotome Parameter werden Häufigkeiten und Prozentwerte angegeben.

## Ergebnisse

### Patientencharakteristika

Insgesamt wurden 226 Patientenvisiten durchgeführt, dokumentiert und ausgewertet. *Das Patientenkollektiv setzte sich aus 170 individuellen Patienten zusammen (58,8* *% Frauen), die im Untersuchungszeitraum ein- bis 6‑mal visitiert wurden *(Tab. 1). Das Durchschnittsalter (± SD) des Patientenkollektivs lag bei 80,9 ± 7,1 Jahren (Spannbreite: 65 bis 101 Jahre). Einen Überblick über die unfallchirurgischen Diagnosen bzw. betroffenen Körperregionen sowie die Komorbiditäten gibt Tab. 1.

**Table 1:** 

Merkmal	Anzahl (*n*)	Anteil (%)^a^
*Geschlecht* (Alter 80,9 ± 7,1 Jahre)		
Weiblich	100	58,8
Männlich	70	41,2
*Betroffene Körperregion bzw. unfallchirurgische Diagnose*		
Schädel	9	5,3
Körperstamm^b^	16	9,4
Obere Extremität^c^	18	10,6
Becken, inkl. Os sacrum	20	11,8
Proximale Femurfraktur	35	20,6
Periprothetische Fraktur	3	1,8
Untere Extremität^d^	45	26,5
Mehr als eine der genannten Körperregionen betroffen	24	14,1
*Patientenkontakte in den Visiten*^*e*^		
Einmal visitiert	136	80,0
2‑mal visitiert	21	12,4
3‑mal visitiert	7	4,1
4‑mal visitiert	4	2,4
5‑mal visitiert	1	0,6
6‑mal visitiert	1	0,6
*Komorbiditäten*		
Arterielle Hypertonie	102	60,0
Koronare Herzerkrankung	19	11,2
Herzinsuffizienz	21	12,4
Periphere arterielle Verschlusskrankheit	10	5,9
Vorhofflimmern	33	19,4
Zustand nach Schlaganfall	7	4,1
Diabetes mellitus Typ 2	33	19,4
Hypothyreose	14	8,2
Chronisch obstruktive Lungenerkrankung	13	7,6
Parkinson-Syndrom	6	3,5
Demenz	21	12,4
Andere Vorerkrankung(en)	118	69,4
*Delirantes Syndrom bei Visite*		
Ja	35	15,5
Nein	191	84,5
*Nierenfunktion (eGFR nach CKD-EPI in [ml/min]/1,73 m*^*2*^)^f^		
≥90 (G1)	19	8,4
60–89 (G2)	98	43,4
45–59 (G3a)	42	18,6
30–44 (G3b)	39	17,3
15–29 (G4)	21	9,3
<15 (G5)	7	3,1

^a^Werte auf eine Nachkommastelle gerundet

^b^Hals‑, Brust‑, Lendenwirbelsäule, Sternum, Rippen

^c^Inklusive Scapula und Clavicula

^d^Mit Ausnahme proximaler Femurfrakturen und periprothetischer Frakturen

^e^Bezogen auf alle 29 Visitentage im Zeitraum 31.05.2019–13.12.2019

^f^Einteilung der Nierenfunktion anhand der geschätzten glomerulären Filtrationsrate (*eGFR*) gemäß der Formel der Chronic Kidney Disease Epidemiology Collaboration (CKD-EPI) in die Stadien G1–G5 nach Kidney Disease Improving Global Outcomes (KDIGO)

Siebzig Prozent des Patientenkollektivs waren zum Zeitpunkt der Visite bereits operiert worden, die übrigen 30 % wurden im Verlauf operiert oder wurden konservativ behandelt. Die bereits operierten Patienten wurden im Median am 4. postoperativen Tag visitiert (Mittelwert ± SD: 6,2 ± 5,5 Tage postoperativ). Bei 15 % der Patienten konnte eine akute Verwirrtheit im Sinne eines deliranten Syndroms detektiert werden. Knapp die Hälfte der Delirien (49 %) entwickelte sich auf dem Boden einer vorbestehenden Demenz.

Die geschätzte glomeruläre Filtrationsrate (eGFR) nach der Formel der Chronic Kidney Disease Epidemiology Collaboration (CKD-EPI) [16] lag im Durchschnitt bei 60 ± 24 (ml/min)/1,73 m^2^. Die Verteilung der Patienten auf die Nierenfunktionskategorien G1–G5 nach Kidney Disease Improving Global Outcomes (KDIGO) zeigt Tab. 1.

### Empfehlungen

Im Rahmen der 226 Patientenkontakte wurden insgesamt 687 Handlungsempfehlungen ausgesprochen. Diese verteilten sich auf 65,6 % pharmakologische Empfehlungen (PE) und 34,4 % allgemeine Empfehlungen (AE) (Tab. 2). Pro Patientenkontakt wurden 3,0 ± 1,9 Handlungsempfehlungen ausgesprochen (2,0 ± 1,4 PE; 1,0 ± 1,3 AE).

**Table 2:** 

Kategorie	Anzahl (*n*)	Anteil (%)^a^
*Alle Empfehlungen*	687	100
Pharmakologische Empfehlungen	451	65,6
Allgemeine Empfehlungen	236	34,4
*Pharmakologische Empfehlungen*	451	100
Medikament absetzen	137	30,4
Medikament pausieren	23	5,1
Medikament (wieder)ansetzen	86	19,1
Dosisreduktion	49	10,9
Dosissteigerung	28	6,2
Medikament umstellen	25	5,5
Überprüfung der Indikation bzw. der Dosierung	24	5,3
Antiinfektive Therapie	77	17,1
Andere	2	0,4
*Allgemeine Empfehlungen*	236	100
Physikalische therapeutische Maßnahmen	22	9,3
Intravenöse Flüssigkeitssubstitution	11	4,7
Ernährungsmedizinische Empfehlungen	4	1,7
Therapie mit Blutprodukten	2	0,8
Delirmanagement	15	6,4
Diagnostik	84	35,6
Konsiluntersuchungen	6	2,5
Medizinische Fremdkörper	58	24,6
Maßnahmen nach Abschluss des stationären Aufenthalts	20	8,5
Empfehlungen für den Arztbrief	12	5,1
Andere	2	0,8

^a^Werte auf eine Nachkommastelle gerundet

### Pharmakologische Empfehlungen

**Figure 1:**
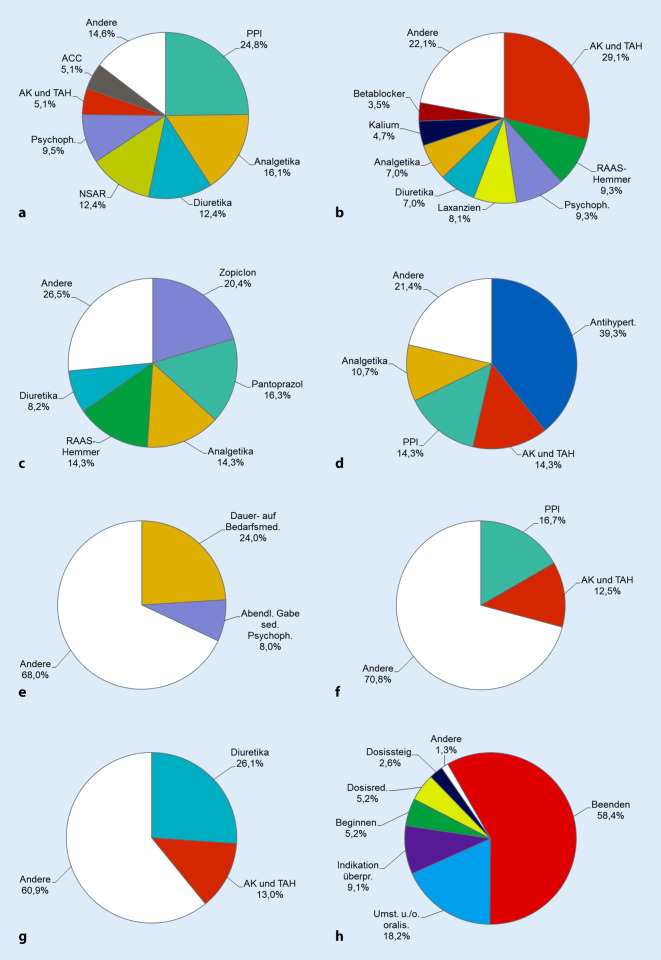

Die PE ließen sich in folgende Kategorien unterteilen (Tab. 2; Abb. 1):

- Medikament absetzen: *Hauptgründe für das Absetzen waren eine fehlende Indikation (v.* *a. bei Protonenpumpeninhibitoren (PPI)) sowie Doppelverordnungen*.
- Medikament ansetzen bzw. nach Pausierung wiederansetzen: Am häufigsten wiederangesetzt wurden Antikoagulanzien (AK) und Thrombozytenaggregationshemmer (TAH) nach erfolgter Operation.
- Dosisreduktion: Zopiclon in einer Dosierung von 7,5 mg ist ein Medikament der PRISCUS-Liste und sollte bei geriatrischen Patienten, falls eine medikamentöse Schlafinduktion erforderlich ist, in der halbierten Dosis von 3,75 mg eingesetzt werden [8]. *Wird Pantoprazol prophylaktisch, z.* *B. zur Gastroprotektion bei gleichzeitiger Einnahme von nichtsteroidalen Antirheumatika (NSAR) eingesetzt, genügen in der Regel 20* *mg/d* [9].
- Dosiserhöhung: Antihypertensiva wurden gesteigert, falls es im Rahmen des stationären Aufenthalts zu einem Blutdruckanstieg kam. Wird Apixaban zur Prophylaxe von Schlaganfällen und systemischen Embolien bei Patienten mit Vorhofflimmern eingesetzt, so richtet sich die Dosierung nach dem Alter, dem Körpergewicht und dem Serum-Kreatinin-Wert des Patienten [9]. Treffen mindestens 2 der 3 Faktoren Alter ≥80 Jahre, Körpergewicht ≤60 kg und Serum-Kreatinin-Wert ≥1,5 mg/dl zu, so sollte eine Dosis von 2‑mal 2,5 mg/d verwendet werden, in allen anderen Fällen 2‑mal 5 mg/d. *In schweren Fällen einer gastroösophagealen Refluxkrankheit oder eines Magen- oder Duodenalulkus können für einen begrenzten Zeitraum (z. B. 4 bis 8 Wochen) 2‑mal 40 mg Pantoprazol (statt einmal 40 mg)/d verabreicht werden*. In einem Fall wurden aufgrund eines Übertragungsfehlers bei einem Patienten mit einer Erkrankung aus dem rheumatischen Formenkreis nur 0,5 mg Prednisolon/d anstelle der vorgesehenen 5 mg verabreicht. Die plötzliche Reduktion der Prednisolondosis um 90 % hätte zu einer akuten Nebennierenrindeninsuffizienz mit u. U. lebensbedrohlichen Konsequenzen führen können [4], weshalb die Prednisolondosis wieder auf das Ausgangsniveau angehoben wurde.
- Medikament umstellen: *Hierzu zählten die Umstellung einer Dauer- auf eine Bedarfsmedikation, z.* *B. für Metamizol oder Pantoprazol, sowie die Umstellung einer morgendlichen Gabe sedierend wirkender Psychopharmaka (Pipamperon, Amitriptylin) auf eine Gabe zur Nacht, um Tagesmüdigkeit zu vermeiden*. Aktivierende Psychopharmaka wie Fluoxetin sollten hingegen morgens statt abends verabreicht werden [13], um den Nachtschlaf nicht zu beeinträchtigen. Dasselbe gilt für Diuretika, die ebenfalls nur tagsüber, nicht aber am Abend oder zur Nacht verabreicht werden sollten, da dies zu Stürzen beim nächtlichen Toilettengang führen kann [12]. *Weitere Beispiele in dieser Kategorie waren die Umstellung des Opioidanalgetikums Tramadol auf Tilidin–Naloxon aufgrund der seltener auftretenden Übelkeit sowie die Umstellung des konventionellen Antipsychotikums (KAP) Pipamperon auf das atypische Antipsychotikum (AAP) Quetiapin bei einem Patienten mit M. Parkinson*. AAP führen im Vergleich zu KAP seltener zu extrapyramidal-motorischen Störungen wie Rigor und sind daher bei älteren Patienten prinzipiell besser geeignet als KAP [13].
- Überprüfung der Indikation bzw. der Dosierung: *Die Dosis renal eliminierter Pharmaka wie Spironolacton, Xipamid und Allopurinol muss an eine eingeschränkte Nierenfunktion angepasst werden.*
- Medikament pausieren: Gründe für das Pausieren von Diuretika waren eine eingeschränkte Nierenfunktion oder eine Exsikkosegefahr bei hohen Außentemperaturen. Gründe für das Pausieren von AK und TAH waren eine erhöhte Sturzgefahr mit Blutungsrisiko sowie Blutungskomplikationen.
- Empfehlungen zur antiinfektiven Therapie.

### Allgemeine Empfehlungen

Die AE ließen sich wie folgt differenzieren:

- Diagnostische Maßnahmen: Laborkontrollen, apparative Untersuchungen (z. B. Röntgen, Sonographie), Maßnahmen zur Infektfokussuche (z. B. Urin- und Stuhluntersuchungen), therapeutisches Drugmonitoring, Messung von Vitalparametern, Ein- und Ausfuhrkontrolle.
- Empfehlungen zu medizinischen Fremdkörpern: *Am häufigsten wurde geraten, einen PVK, ZVK oder transurethralen Harnblasenkatheter zu entfernen.*
- Physikalische therapeutische Maßnahmen: *intensivierte Flüssigkeitszufuhr per os, Physiotherapie, Atemgymnastik, Inhalation mit 0,9-prozentiger NaCl-Lösung zur Sekretolyse.*
- Maßnahmen nach Abschluss des stationären Aufenthalts: *In dieser Kategorie dominierte die geriatrische Frührehabilitation (OPS (Operationen- und Prozedurenschlüssel) 8‑550).*
- Management deliranter Patienten: konsequente Anwendung von Seh- und Hörhilfen, enge Betreuung durch das ärztliche und pflegerische Personal, Anwesenheit von Angehörigen, Reorientierungsmaßnahmen (z. B. Anbieten einer Tageszeitung, Aufstellen einer Uhr/eines Kalenders in der unmittelbaren Patientenumgebung) [10].
- Empfehlungen für den Arztbrief: *Ein während des stationären Aufenthalts aufgetretenes delirantes Syndrom sollte als Diagnose im Arztbrief erfasst werden*.
- Intravenöse Flüssigkeitssubstitution.
- Konsiluntersuchungen bei anderen Fachdisziplinen.
- Ernährungsmedizinische Empfehlungen: Bei geriatrischen Patienten sollte Wert auf eine ausreichende Eiweißzufuhr zu Prophylaxe bzw. Therapie einer Sarkopenie gelegt werden.
- Therapie mit Blutprodukten.

## Diskussion

Besonders hervorzuheben ist der interdisziplinäre und interprofessionelle Charakter unserer alterstraumatologischen Visite, da neben Ärzten verschiedener Fachdisziplinen Pflegekräfte und Case Manager an unserer Visite teilnahmen.

Erstaunlicherweise konnten ca. 60 % mehr Medikamente abgesetzt als neu bzw. wieder angesetzt werden. Dies zeigt, dass eine kritische Indikationsstellung und das Vermeiden von Doppelverordnungen effektive Maßnahmen zur Reduktion einer inadäquaten Polypharmazie darstellen. Die Medikamentenklasse, für die sich in unserer Untersuchung am seltensten eine eindeutige Indikation feststellen ließ, waren PPI. Dies deckt sich mit den Ergebnissen einer Studie von Heidelbaugh et al., in der in 36,1 % der Fälle PPI ohne klinisch eindeutige Indikation verschrieben wurden, allerdings im ambulanten Bereich [6]. Werden PPI für einen begrenzten Zeitraum eingenommen, handelt es sich in der Regel um gut verträgliche Medikamente. *Problematisch ist insbesondere die unkritische Langzeitapplikation von PPI, welche mit einem erhöhten Risiko für Pneumonien, gastrointestinale Infektionen und Osteoporose vergesellschaftet ist* [7].

Interessanterweise bewegte sich das Verhältnis von Dosisreduktionen zu Dosissteigerungen in unserer Untersuchung in einer ähnlichen Größenordnung wie das oben beschriebene Verhältnis von Medikamente absetzen zu Medikamente (wieder)ansetzen. Insgesamt konnten 75 % mehr Dosisreduktionen als -steigerungen vorgenommen werden. Werden Antihypertensiva zu hoch dosiert, kann es bei geriatrischen Patienten zu gefährlichen Hypotonien kommen [12], wodurch sich die Sturz- und Frakturgefahr dramatisch erhöht. Daher sind die in den jeweiligen Fachinformationen angegebenen Tageshöchstdosen unbedingt zu beachten. Bei gleichzeitiger Anwendung von Amlodipin und Simvastatin kann es zu einer klinisch relevanten Medikamenteninteraktion kommen [17]. Die Hemmung des Cytochrom-P450(CYP) -Isoenzyms 3A4 durch Amlodipin führt zu einer Erhöhung der Plasmakonzentration des CYP3A4-Substrats Simvastatin mit der Gefahr einer Myopathie bis hin zur Rhabdomyolyse. Unter Komedikation mit Amlodipin sollte die maximale Simvastatintagesdosis daher 20 mg nicht überschreiten [9].

Medikamentenumstellungen betrafen einerseits Präparatewechsel aufgrund von Nebenwirkungen oder Kontraindikationen, wie z. B. bei der Umstellung von Metamizol auf Paracetamol bei Patienten mit Leukopenie oder anderen Blutbildungsstörungen. Andererseits mussten zirkadiane Aspekte der Pharmakotherapie berücksichtigt werden, wie die Vermeidung einer abendlichen Gabe von Diuretika oder einer morgendlichen Gabe sedierend wirkender Psychopharmaka (z. B. Amitriptylin, Quetiapin, Pipamperon). Viele Psychopharmaka, insbesondere Benzodiazepine, besitzen ein delirogenes Potenzial und sollten bei geriatrischen Patienten äußerst restriktiv eingesetzt werden.

Angesichts des hohen Durchschnittsalters von knapp über 80 Jahren wenig überraschend war der Befund, dass 92 % unseres Patientenkollektivs eine eingeschränkte Nierenfunktion (eGFR <90 [ml/min]/1,73 m^2^) aufwiesen. *Dies ist insofern bedeutsam, als dass zahlreiche Medikamente wie Inhibitoren des Renin-Angiotensin-Aldosteron-Systems (RAAS-Hemmer), direkte orale Antikoagulanzien, Metformin, Allopurinol und Pregabalin einer Dosisanpassung bei eingeschränkter Nierenfunktion bedürfen*. Darüber hinaus sollten bei eingeschränkter Nierenfunktion nephrotoxische Medikamente wie NSAR nach Möglichkeit vermieden werden. Besondere Bedeutung erlangt in diesem Kontext der „triple whammy“, die (risikoreiche) gleichzeitige Anwendung eines NSAR, eines RAAS-Hemmers und eines Diuretikums [3], die eine Nierenfunktionsverschlechterung bis hin zum akuten Nierenversagen nach sich ziehen kann.

Ungefähr jede fünfte PE betraf die Therapie mit Antiinfektiva. Erstaunlicherweise wurde die Empfehlung, eine antiinfektive Therapie zu beenden, mehr als 11-mal häufiger ausgesprochen als die Empfehlung, eine antiinfektive Therapie neu zu beginnen. Durch die Expertise des ABS-Teams wurde beispielweise die Gabe oraler Cephalosporine aufgrund ihrer unzureichenden oralen Bioverfügbarkeit nahezu vollständig eingestellt. Eine rationale antiinfektive Therapie beugt der Entwicklung von Antibiotikaresistenzen vor. Ein ABS-Programm kann hierzu einen wertvollen Beitrag leisten.

Ein Krankenhausaufenthalt erfordert oftmals die Anlage medizinischer Fremdkörper wie PVK und/oder ZVK sowie ggf. eines transurethralen Harnblasenkatheters. Im Rahmen unserer Visite wurde standardmäßig überprüft, ob für medizinische Fremdkörper (weiterhin) eine Indikation bestand. Erhielt ein Patient beispielweise nur Flüssigkeit, aber keine Medikamente über einen PVK und konnte ausreichende Flüssigkeitsmengen peroral zu sich nehmen, so wurde die Empfehlung ausgesprochen, den PVK zu entfernen, um die Anzahl potenzieller Eintrittspforten für Krankheitserreger in den Körper zu minimieren. Zeigten sich lokale Entzündungszeichen, so wurden die betroffenen Fremdkörper umgehend entfernt. Durch diese einfachen Maßnahmen können katheterassoziierte Infektionen, eine der wichtigsten Ursachen krankenhausbedingter Morbidität und Mortalität, verhindert werden [1, 18].

*Durch die routinemäßige Überprüfung der Orientierung und Vigilanz kann die Entwicklung eines postoperativen Delirs erkannt und können frühzeitig entsprechende Gegenmaßnahmen eingeleitet werden.* In unserem Patientenkollektiv wurde in 15 % eine akute Verwirrtheit im Sinne eines deliranten Syndroms festgestellt. Dies liegt im unteren Bereich der in der Literatur angegebenen Werte von 11–42 % bei hospitalisierten Patienten über 65 Jahren [19]. Während in unserer Untersuchung bei 35 Patienten ein Delir vorlag, wurden insgesamt nur 15 delirspezifische AE empfohlen. Diese Diskrepanz lässt sich möglicherweise dadurch erklären, dass Maßnahmen wie die Sicherstellung einer adäquaten Flüssigkeitszufuhr und das Absetzen delirogener Medikamente in andere Empfehlungskategorien eingruppiert wurden. Hinzu kommt, dass an der Medizinischen Hochschule Hannover regelmäßig Schulungen zum Thema Delir stattfinden und daher ggf. bereits vor dem Zeitpunkt der Visite Gegenmaßnahmen eingeleitet worden waren. *Häufig bleibt unklar, ob ein Patient bei vorherigen Operationen ein Delir erlitten hat*. Daher sollte das Delir bzw. delirante Syndrom immer im Entlassungsbrief aufgeführt werden.

### Limitationen

Limitationen unserer Untersuchung ergeben sich aus dem monozentrischen Design sowie dem Fehlen einer Kontrollgruppe. *Daher bleibt offen, ob durch unsere Visite tatsächlich Komplikationen verhindert werden konnten. Schulungsmaßnahmen für das chirurgische Personal im Hinblick auf die Arzneimitteltherapie bei älteren Patienten fanden nicht statt. Der Fokus unserer Arbeit bestand darin, die gängige Standardbehandlung an unserer Klinik zu evaluieren und Empfehlungen zur Optimierung abzuleiten. Unsere Arbeit sollte daher als Pilotstudie zur Etablierung eines umfassenderen alterstraumatologischen Konzepts verstanden werden.*

*Da für unsere Visite nur ein Zeitfenster von ca. 2* *h einmal wöchentlich zur Verfügung stand, konnten Vigilanz, Kognition, Mobilität etc. nur klinisch-orientierend geprüft werden. Geriatrische Assessments im eigentlichen Sinn kamen nicht zum Einsatz.*

*Die Vorerkrankungen wurden nur retrospektiv dem Aufnahmebefund bzw. der Krankenakte entnommen*. Des Weiteren wurde die Art der operativen Versorgung nicht erfasst. Nichtsdestotrotz gehen wir davon aus, dass unser Patientenkollektiv repräsentativ für eine deutsche unfallchirurgische Akutklinik mit Schwerpunktversorgung ist. Hervorzuheben ist, dass für unsere Untersuchung keine expliziten Ausschlusskriterien bestanden und dass alle vorab ausgewählten Patienten auch tatsächlich visitiert wurden (keine Drop-outs).

*Eine weitere Limitation könnte darin bestehen, dass für kleinere bzw. nichtuniversitäre Kliniken eine interdisziplinäre alterstraumatologische Visite aufgrund des hohen Zeit- und Personalaufwands schwierig umsetzbar ist. Daher sind unsere Ergebnisse nicht uneingeschränkt auf andere Einrichtungen übertragbar.*

## Fazit für die Praxis

- Zur Vermeidung einer Hypotonie mit Sturzgefahr muss die Tageshöchstdosis von Antihypertensiva beachtet werden: je 10 mg für Ramipril und Amlodipin, 32 mg für Candesartan.
- Doppelverordnungen, z. B. zweier verschiedener Opioidanalgetika oder von Ibuprofen plus Paracetamol, sind nach Möglichkeit zu vermeiden.
- Wird Pantoprazol in prophylaktischer Indikation eingesetzt, so genügen in der Regel 20 mg/d.
- Unter Komedikation mit Amlodipin beträgt die maximale Simvastatintagesdosis 20 mg.
- Bei Schlafstörungen sollte Zopiclon (wenn überhaupt) in einer reduzierten Dosis von 3,75 mg eingesetzt werden.
- Ein (postoperatives) delirantes Syndrom sollte als Diagnose im Entlassungsbericht aufgeführt werden.
- Auf die Gabe von Cefuroxim per os sollte aufgrund der geringen Bioverfügbarkeit verzichtet werden.
